# The clarithromycin-binding proteins NIPSNAP1 and 2 regulate cytokine production through mitochondrial quality control

**DOI:** 10.1038/s41598-024-52582-7

**Published:** 2024-01-29

**Authors:** Soh Yamamoto, Noriko Ogasawara, Yukari Mitsuhashi, Kenichi Takano, Shin-ichi Yokota

**Affiliations:** 1 Department of Microbiology, Sapporo Medical University School of Medicine, Sapporo, Japan; 2 Department of Otolaryngology-Head and Neck Surgery, Sapporo Medical University School of Medicine, Sapporo, Japan

**Keywords:** Cell biology, Chemical biology, Immunology, Microbiology

## Abstract

The mechanism underlying the anti-inflammatory effect of macrolide antibiotics, such as clarithromycin (CAM), remains to be clarified. The CAM-binding proteins 4-nitrophenylphosphatase domain and non-neuronal synaptosomal associated protein 25 (SNAP25)-like protein homolog (NIPSNAP) 1 and 2 are involved in the immune response and mitochondrial homeostasis. However, the axis between CAM-NIPSNAP-mitochondria and Toll-like receptor (TLR) and their molecular mechanisms remain unknown. In this study, we sought to elucidate the relationship between mitochondrial homeostasis mediated by NIPSNAP1 and 2 and the immunomodulatory effect of CAM. NIPSNAP1 or 2 knockdown (KD) by RNA interference impaired TLR4-mediated interleukin-8 (IL-8) production. Similar impairment was observed upon treatment with mitochondrial function inhibitors. However, IL-8 secretion was not impaired in NIPSNAP1 and 2 individual knockout (KO) and double KO (DKO) cells. Moreover, the oxygen consumption rate (OCR) in mitochondria measured using a flex analyzer was significantly reduced in NIPSNAP1 or 2 KD cells, but not in DKO cells. CAM also dose-dependently reduced the OCR. These results indicate that CAM suppresses the IL-8 production via the mitochondrial quality control regulated by temporary functional inhibition of NIPSNAP1 and 2. Our findings provide new insight into the mechanisms underlying cytokine production, including the TLR-mitochondria axis, and the immunomodulatory effects of macrolides.

## Introduction

The 4-nitrophenylphosphatase domain and non-neuronal synaptosomal associated protein 25 (SNAP25)-like protein homolog (NIPSNAP) family consists of four homologs^1–4^, which localize mainly to mitochondria. Their domain construction is evolutionarily conserved from mammals to bacteria^1^. The genes encoding NIPSNAP1 and 2 are located on chromosomes 22q12 and 7p12, respectively, and those encoding NIPSNAP3 and 4 are located on chromosome 9q31. The tissue localizations, expression levels, and functions of NIPSNAP1/2 and NIPSNAP3/4 differ^1^. These differences are consistent with their homology; there is high homology between NIPSNAP1 and 2 and between NIPSNAP3 and 4. A recent study demonstrated that NIPSNAP1 and 2 are responsible for maintaining mitochondrial homeostasis^3^. Induction of NIPSNAP1 and 2 on the outer mitochondrial membrane (OMM) leads to the clearance of damaged mitochondria by selective autophagy, called mitophagy^3,4^. The function of NIPSNAP1 has been determined in slowly proliferating cells, such as nerve cells, and is associated with the pathogenesis of Parkinson's disease^3^. However, the detailed function of NIPSNAPs in other cells, including airway epithelial cells, is unknown.

Mitochondria are versatile organelles fundamental for life^4^,^5^. Recent research demonstrated that mitochondria act as a "signaling hub" in innate immune signaling pathways. Various signals are relayed in mitochondria and transmitted to the nucleus. During viral infection, mitochondrial antiviral signaling protein (MAVS) plays an important role in receiving signals from pattern recognition receptors and transducing them to the downstream nuclear factor-kappa B (NF-κB) and interferon regulatory factor (IRF) signaling pathways for the production of pro-inflammatory cytokines, chemokines, and interferons^5^. Although the detailed mechanism underlying signal transduction via mitochondria is not fully understood, mitochondria play a crucial role in the immune response to environmental stimuli.

It is well known that 14- and 15-membered macrolide antibacterial agents, including clarithromycin (CAM), temporarily suppress the induction of Toll-like receptor (TLR)-mediated cytokines^6^, such as interleukin-8 (IL-8). Previously, we found that CAM binds to NIPSNAP1 and 2, and suggested that these proteins are involved in IL-8 induction via NF-κB activation upon TLR4 stimulation^7^. In addition, we reported that CAM suppresses respiratory syncytial virus-induced cytokine production by preventing the translocation of transcription factors into the nucleus^8^. However, the suppressive effects of CAM on cytokine production induced by various stimulations remain to be explained.

This study aimed to elucidate the effect of the CAM-binding proteins NIPSNAP1 and 2 on the innate immune response through TLR4-mediated IL-8 production in airway epithelial cells. We investigated the relationship between IL-8 production and mitochondrial function using genetic engineering and chemical approaches. Our study is the first to elucidate the relationship between TLR4-mediated IL-8 production and mitochondrial quality control mediated by NIPSNAPs.

## Results

### NIPSNAP1 or 2 knockdown (KD) suppresses TLR4-mediated IL-8 production in airway epithelial cells

**Figure 1:**
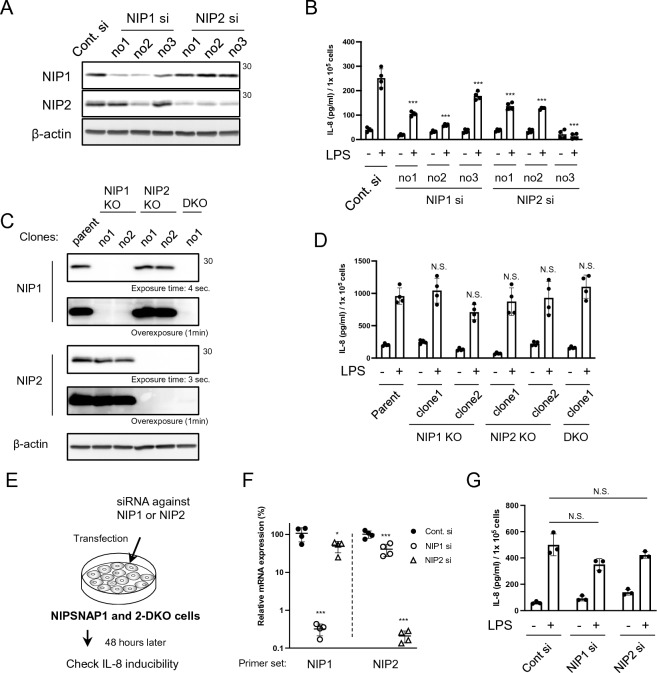
Temporary depletion of NIPSNAPs suppresses LPS-induced IL-8 production. (**A**) Representative WB image (cropped) from three independent experiments of BEAS-2B cells at 48 h after siRNA transfection. Cont. si denotes nontargeting siRNA. siRNAs (no. 1–3) have different anti-sense sequences (details are provided in the “Materials and Methods”). NIP1 and NIP2 denote NIPSNAP1 and 2, respectively. (**B**) IL-8 protein levels in the culture supernatant of NIPSNAP1 or 2 KD BEAS-2B cells were measured by an ELISA. At 48 h after siRNA transfection, cells were stimulated with or without 100 ng/mL LPS for 6 h. The IL-8 protein level was normalized by the cell number. Bars show the mean ± SD of four independent experiments. ***P < 0.001 vs. cells stimulated with LPS and transfected with nontargeting siRNA, one-way ANOVA. (**C**) Detection of NIPSNAP1 and 2 in two clones each of NIPSNAP1 and 2 KO clones, DKO cells, and parental BEAS-2B cells by WB (cropped). (**D**) The normalized IL-8 protein levels in the culture supernatant were measured after incubation with or without 100 ng/mL LPS for 6 h. Bars show the mean ± SD of four independent experiments. N.S., not significant, one-way ANOVA. (**E**) NIPSNAP1 and 2 DKO BEAS-2B cells shown in panel C (right lane) were transfected with siRNA against NIPSNAP1 or 2. (**F** and **G**) At 48 h after siRNA transfection, the mRNA levels of NIPSNAP1 and 2 were quantified by qPCR (**F**). Normalized expression in nontargeting siRNA-transfected cells was set to 100% (closed circles). Open circles and triangles indicate cells transfected with NIPSNAP1- and 2-targeting siRNA, respectively. Primer set means the primer set used to detect each NIPSNAP. Bars show the mean ± SD of four independent experiments. *P < 0.05 and ***P < 0.001, one-way ANOVA vs. Cont. si. DKO cells were incubated with or without 100 ng/mL LPS for 6 h. The normalized IL-8 protein levels in the culture supernatant were measured (**G**). Bars show the mean ± SD of three independent experiments. N.S.; not significant, Student's t-test. Note that WB images were cropped to remove irrelevant areas, and the original images are shown in supplemental Fig. S1.

To investigate whether NIPSNAP1 and 2 are involved in cytokine production, we measured the IL-8 protein levels in the culture supernatant of BEAS-2B cells, a human bronchial epithelial cell line, transfected with three different siRNA sequences targeting NIPSNAP1 or 2 to consider off-target effect of siRNAs (no. 1–3; details are provided in the “Materials and Methods”) (Fig. 1A). IL-8 induction upon lipopolysaccharide (LPS) treatment was significantly suppressed in NIPSNAP1 or 2 KD cells compared with nontargeting siRNA-transfected cells (Fig. 1B). The decrease in IL-8 production was similar in cells transfected with the three different siRNA sequences.

Furthermore, we generated NIPSNAP1 and 2 knockout (KO) and double knockout (DKO) BEAS-2B cells by CRISPR/Cas9 genome editing (Fig. 1C). In contrast with the results obtained using NIPSNAP1 or 2 KD cells, LPS-stimulated IL-8 induction in KO and DKO cells was comparable with that in parental BEAS-2B cells (Fig. 1D). To exclude the possibility of an off-target effect of siRNAs on NIPSNAP1 or 2 KD cells, DKO cells were transfected with siRNA against NIPSNAP1 (no. 1) or 2 (no. 1) (Fig. 1E). mRNA expression of NIPSNAP1 and 2 was decreased by more than 99% (Fig. 1F). Induction of IL-8 by LPS in these cells was comparable with that in nontargeting siRNA-transfected cells (Fig. 1G). Temporary downregulation of NIPSNAP1 or 2 suppressed IL-8 induction; however, the complete deficiency of NIPSNAP1 and 2 did not (Fig. 1A–D). These unexpected observations suggest that acute functional inhibition of NIPSNAP1 or 2 perturbs the induction of IL-8 by LPS.

### Mitochondrial stress reduces IL-8 production

**Figure 2:**
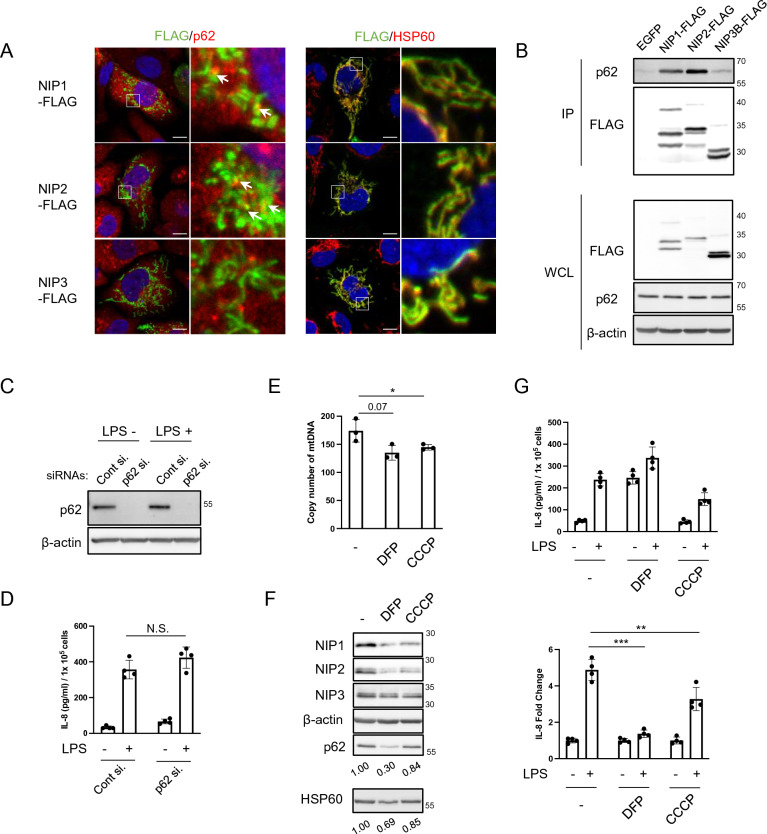
Mitochondria are involved in LPS-induced IL-8 production. (**A**) Co-immunofluorescence imaging of NIPSNAPs (NIP1, 2, and 3; green) and HSP60 (a mitochondrial protein, red) or p62 (red). FLAG-tagged NIPSNAP1-, 2-, and 3-expressing BEAS-2B cells were co-stained with the indicated antibodies. The right columns are enlargements of the boxed regions in the left columns. Arrows in the right columns indicate colocalization of NIPSNAPs and p62. White scale bars in the left columns indicate 10 μm. (**B**) Detection of p62 interacting with NIPSNAPs in BEAS-2B cells by immunoprecipitation and WB (cropped). EGFP-expressing BEAS-2B cells were used as a negative control. IP and WCL indicate immunoprecipitate and whole-cell lysate, respectively. The numbers on the right indicate the molecular weights (kDa) of protein standards. (**C** and **D**) Detection of p62 by WB (**C**: cropped) and the normalized IL-8 level in the culture supernatant (**D**) of p62 KD BEAS-2B cells stimulated with or without 100 ng/mL LPS for 6 h. Bars show the mean ± SD of four independent experiments. N.S., not significant, Student's t-test. (**E**–**G**) BEAS-2B (**E** and **G**) or H1-HeLa (**F**) cells were incubated with DFP (1 mM) or CCCP (20 μM) in the presence of 20 μM Q-VD-OPH, a caspase inhibitor. After incubation for 16 h, mtDNA (**E**) and the indicated proteins (**F**) were detected by quantitative PCR and WB (cropped), respectively. Relative expression levels of p62 and HSP60 normalized by the expression of β-actin are indicated under the WB images. (**G**) Normalized IL-8 levels (top) and induction rates (bottom) upon treatment with 100 ng/mL LPS for 6 h were quantified by an ELISA. Bars show the mean ± SD of three (for mtDNA measurement) and four (for IL-8 measurement) independent experiments. *P < 0.05, **P < 0.01, and ***P < 0.001; Student's t-test. Note that WB images were cropped to remove irrelevant areas, and the original images are shown in supplemental Fig. S1.

We previously reported that NIPSNAP1 and 2 interact with p62^2^, and NIPSNAP1 or 2 KD impairs NF-κB activation upon LPS stimulation^7^. p62 is involved in NF-κB activation^9^. Immunofluorescence analysis showed that NIPSNAP1 and 2 partly colocalized with p62 as puncta in the cytoplasm under normal conditions (Fig. 2A). NIPSNAP3, a homolog of NIPSNAP1 and 2, mostly colocalized with heat shock protein 60 (HSP60) in a typical mitochondrial mesh structure, but not with p62. These interactions were also examined by co-immunoprecipitation analysis (Fig. 2B). The results were consistent with those of immunofluorescence analysis. Next, we examined whether the interaction between p62 and NIPSNAP1 and 2 contributes to IL-8 production. However, p62 KD did not alter IL-8 induction by LPS (Fig. 2C,D).

Mitochondria act as a signaling hub under stress conditions, such as microbial infection^5^, and NIPSNAP1 and 2 are involved in mitochondrial quality control^3^. To examine IL-8 production upon reduction of mitochondrial function, we treated cells with deferiprone (DFP)^10^, an iron chelator, and *m*-chlorophenylhydrazone (CCCP), a mitochondrial uncoupler^11^. Cells were co-treated with Quinoline-Val-Asp-Difluorophenoxymethylketone (Q-VD-OPH) to prevent apoptosis induced by DFP and CCCP. These inhibitors induced degradation of p62, NIPSNAP1, NIPSNAP2, and HSP60, but not of NIPSNAP3, suggesting that bulk autophagy or mitophagy was induced, resulting in a reduced copy number of mitochondrial DNA (mtDNA) (Fig. 2E,F). These mitochondrial stress conditions significantly suppressed IL-8 induction by LPS (Fig. 2G). These results indicate that mitochondria act as a signaling hub in TLR4-mediated IL-8 production.

### KD of NIPSNAP1 or 2 causes mitochondrial dysfunction, but DKO of NIPSNAP1 and 2 does not

**Figure 3:**
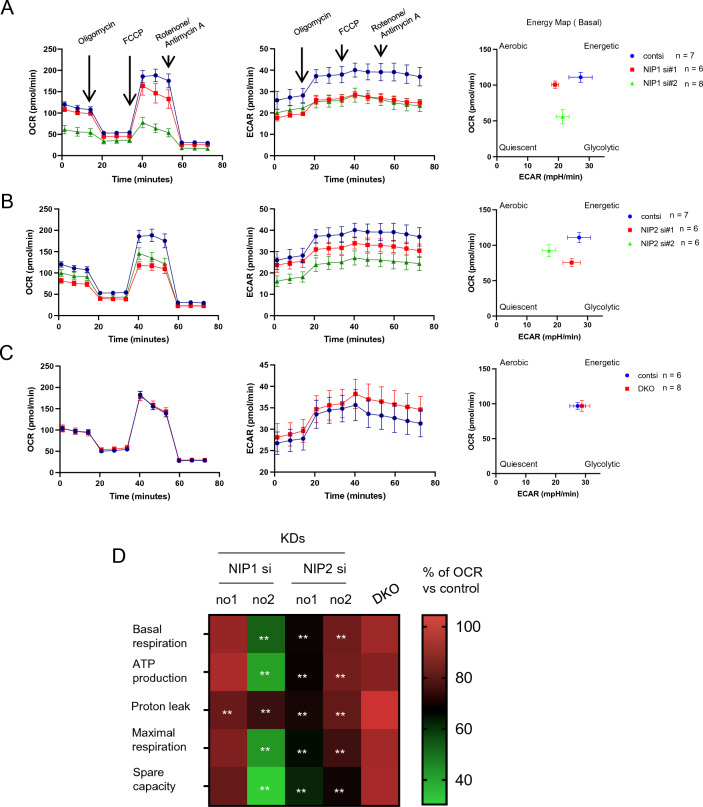
The mitochondrial respiratory rate is decreased upon KD of NIPSNAP1 or 2, but not upon DKO of NIPSNAP1 and 2. (**A**–**C**) Real-time monitoring of the OCR (left panel) and ECAR (middle panel) in NIPSNAP1 (NIP1, A) or NIPSNAP2 (NIP2, **B**) KD BEAS-2B cells, and DKO BEAS-2B cells (**C**). An energy map (right panel) was created at the basal level from the second measurement. Oligomycin (a complex V inhibitor), FCCP (a mitochondrial uncoupler), and a rotenone/antimycin A mixture (respiratory chain inhibitors) were injected at the indicated time points. The numbers (n) indicate the numbers of measurements. (**D**) A heatmap of the bioenergetic profiles of NIPSNAP1 or 2 KD cells, and DKO cells. Each parameter was calculated from the OCR measurements shown in panels (**A**–**C**). For comparison, the values in nontargeting siRNA-transfected or WT (parental cells of DKO) cells were set to 100%. **P < 0.01, Student’s t-test.

To elucidate the effect of NIPSNAP1 and 2 on mitochondrial quality control, we measured the oxygen consumption rate (OCR) in mitochondria using a Flex analyzer. Moreover, the extracellular acidification rate (ECAR) was also monitored. Transfection of siRNAs targeting NIPSNAP1 (no. 1 and 2) or NIPSNAP2 (no. 1 and 2) significantly decreased the OCR compared with transfection of control siRNA (Fig. 3A,B left, and D). By contrast, the ECAR was decreased in both NIPSNAP1 KD and NIPSNAP2 KD (no. 2) cells, but not in NIPSNAP2 KD (no. 1) cells (Fig. 3A,B middle). Metabolism was lower in these KD cells than in control siRNA-transfected cells (Fig. 3A,B right). However, the OCR, the ECAR, and cellular metabolism did not differ between DKO and wild-type (WT) cells (Fig. 3C). We verified and visualized the mitochondrial bioenergetic profiles as a heatmap (Fig. 3D). This showed that KD of NIPSNAP1 or 2 significantly decreased mitochondrial function. However, all mitochondrial functions tested were unaffected in DKO cells, and transfection of NIPSNAP1-targeting siRNA no. 1 did not change the % of OCR except for proton leak. These results suggest that the healthy mitochondrial function in DKO cells is due to the functioning of an alternative pathway.

**Figure 4:**
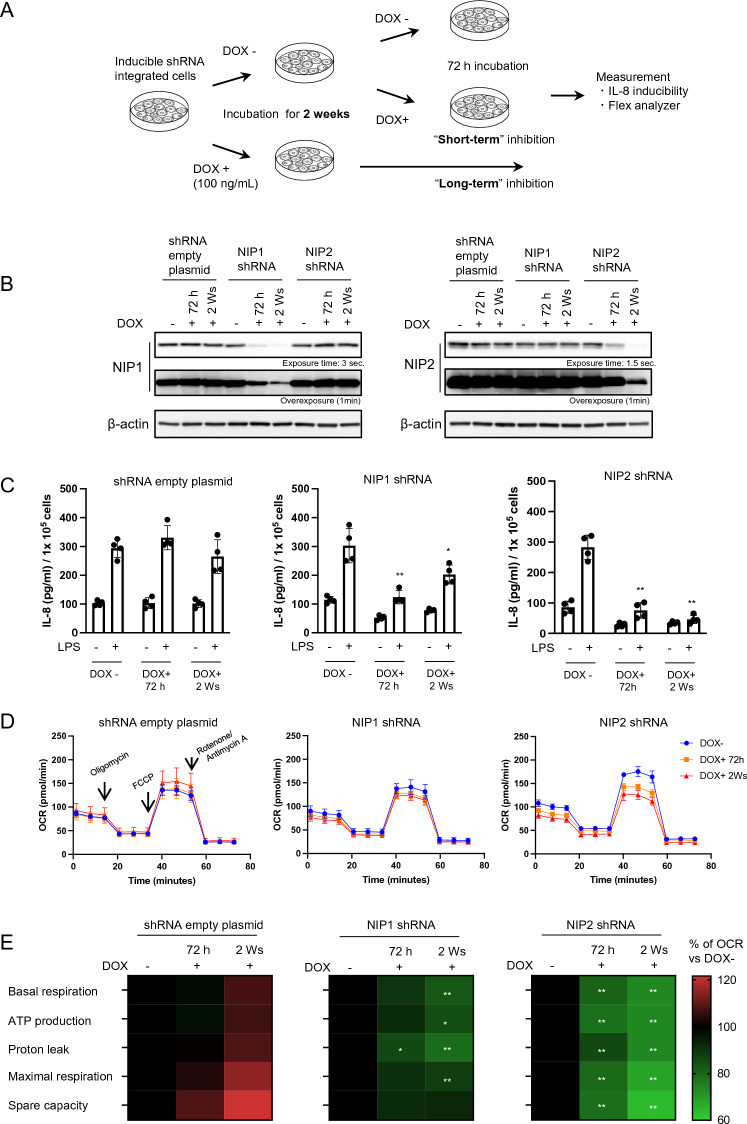
Long-term KD of NIPSNAP1 and 2 suppresses LPS-induced IL-8 production and mitochondrial functions. (**A**) Summary of the experimental scheme. Tetracycline-inducible shRNA-integrated BEAS-2B cells were incubated for 2 weeks with or without 100 ng/mL DOX. Short-term KD of NIPSNAP1 and 2 was performed by incubation with DOX for 72 h. (**B**) Detection of NIPSNAP1 and 2 by WB (cropped). 72 h and 2 Ws mean cells incubated with DOX for 72 h and 2 weeks, respectively. (**C**) Cells were stimulated with or without 100 ng/mL LPS for 6 h. Normalized IL-8 protein levels in the culture supernatant of NIPSNAP1 or 2 KD BEAS-2B cells were measured by an ELISA. Bars show the mean ± SD of four independent experiments. *P < 0.05 and **P < 0.01 vs. cells stimulated with LPS without DOX, Student’s t-test. (**D**) Real-time monitoring of the OCR. The left, middle, and right panels indicate control (non-shRNA), NIPSNAP1 shRNA-expressing, and NIPSNAP2 shRNA-expressing BEAS-2B cells, respectively. Blue, orange, and red lines mean without DOX, incubated for 72 h with DOX, and incubated for 2 weeks with DOX, respectively. (**E**) Heatmaps of the bioenergetic profiles of control (non-shRNA), NIPSNAP1 shRNA-expressing, and NIPSNAP2 shRNA-expressing BEAS-2B cells. Each parameter was calculated from the OCR measurements shown in panel D. For comparison, the value obtained without DOX was set to 100%. *P < 0.05 and **P < 0.01, Student’s t-test. Note that WB images were cropped to remove irrelevant areas, and the original images are shown in supplemental Fig. S1.

To determine whether the phenotype differs depending on the duration of inhibition, NIPSNAP1 and 2 were knocked out for 2 weeks (assumed to mimic the KO condition) using a tetracycline-inducible short hairpin RNA (shRNA) system (Fig. 4A). Furthermore, we measured IL-8 inducibility and mitochondrial function upon short-term KD after incubation with doxycycline (DOX) for 72 h. NIPSNAP1 and 2 proteins were downregulated by shRNA induction. Induction of shRNA for 2 weeks led to extremely low levels of NIPSNAP1 and 2 protein expression (KD efficiency of NIPSNAP1 and 2 was 95.3% and 94.8%, respectively) compared with induction of shRNA for 72 h (KD efficiency of NIPSNAP1 and 2 was 82.1% and 52.8%, respectively) (Fig. 4B). LPS-induced IL-8 secretion was significantly reduced regardless of the duration of KD (Fig. 4C). The OCR in mitochondria and mitochondrial bioenergetic profiles indicated that mitochondrial function was decreased (Fig. 4D,E). Notably, mitochondrial function was lower in long-term KD cells (i.e., shRNA induced for 2 weeks) than in short-term KD cells (i.e., shRNA induced for 72 h). These results indicate that NIPSNAP1 and 2 downregulation, regardless of the KD period, reduces IL-8 production through mitochondrial dysfunction.

### CAM, a compound that binds to NIPSNAP1 and 2, suppresses mitochondrial functions

**Figure 5:**
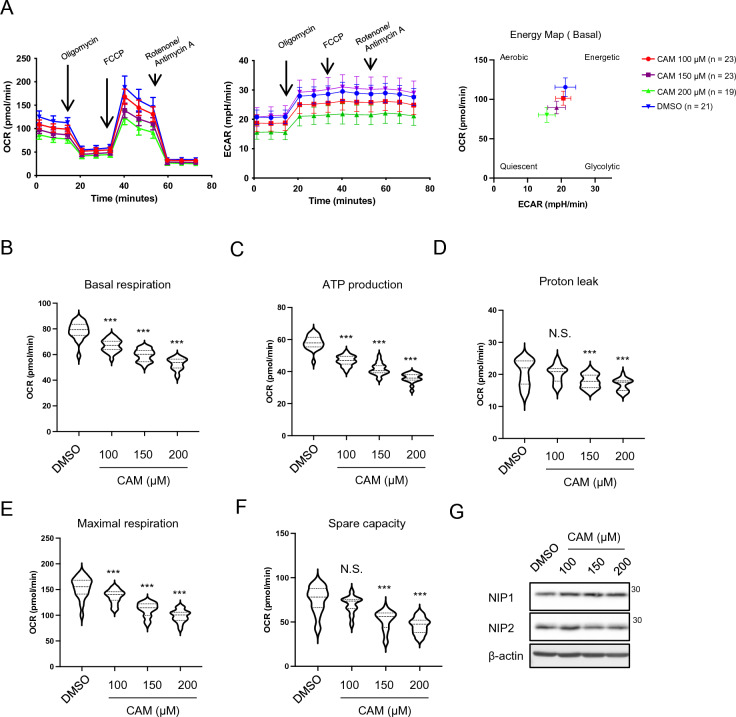
Clarithromycin modulates mitochondrial functions and bioenergetic homeostasis. (**A**) Real-time monitoring of the OCR (left panel) and ECAR (middle panel) in BEAS-2B cells treated with 0, 100, 150, or 200 μM CAM for 22 h. An energy map (right panel) was created using the second measurement. (**B**–**F**) Violin plots of basal respiration (**B**), ATP production (**C**), proton leak (**D**), maximal respiration (**E**), and spare capacity (**F**). (**G**) Detection of NIPSNAP1 and 2 in BEAS-2B cells treated with 0, 100, 150, or 200 μM CAM for 24 h by WB (cropped). ***P < 0.001, one-way ANOVA; N.S., not significant. Note that WB images were cropped to remove irrelevant areas, and the original images are shown in supplemental Fig. S1.

Finally, we investigated the effect of CAM on mitochondrial function. Similar to the effects of NIPSNAP1 or 2 KD, CAM decreased the OCR and ECAR in a dose-dependent manner (Fig. 5A left and middle). In addition, CAM reduced cellular metabolism (Fig. 5A right). Bioenergetic profiles were shown as violin plots (Fig. 5B–F). CAM treatment significantly decreased all profiles. On the other hand, CAM treatment did not affect protein expression of NIPSNAP1 or 2 (Fig. 5G).

## Discussion

We revealed that depletion of NIPSNAP1 or 2 by RNA interference decreased mitochondrial function, resulting in suppression of TLR4-mediated IL-8 production. Moreover, CAM, which binds to NIPSNAP1 and 2^7^, reduced mitochondrial activity in a dose-dependent manner. Our results demonstrate that the well-known immunomodulatory effect of CAM is partly mediated by transient functional inhibition of mitochondria via NIPSNAP1 and 2.

The discrepancy between the effects of NIPSNAP1 or 2 KD (by siRNA or shRNA) and KO might be explained by the results of functional analysis of mitochondria (Figs. 3 and 4). Inhibition of NIPSNAP1 or 2 by RNA interference significantly reduced the bioenergetic profiles compared with transfection of nontargeting siRNA or non-shRNA-induced cells (Figs. 3D and 4E). RNA interference sometimes has an off-target effect. In this study, we used three different siRNAs and shRNA for KD experiments. Although a difference in IL-8 productions was observed between each siRNA, all KD cells consistently showed less production compared with nontargeting siRNA-transfected cells. In addition, siRNA transfection of DKO cells did not affect IL-8 production. Therefore, the possibility of off-target effects on mitochondrial function and IL-8 production was excluded. Our results indicate that NIPSNAP1 and 2 preserve mitochondrial quality control, which is supported by the study by Abudu et al.^3^. Mitochondria are the powerhouse of the cell; they generate ATP via oxidative phosphorylation. Thus, mitochondrial quality is extremely important^4^. Induction of NIPSNAP1 and 2 on the OMM directs selective mitochondrial degradation^3^, followed by release of inflammatory cytokines^12^. The phenotypic difference between KD and KO cells could be explained by the presence of an alternative factor that can compensate for NIPSNAP1 and 2 functions in KO cells, resulting in maintenance of mitochondrial quality. Therefore, the OCR and LPS-induced IL-8 production were comparable between KO and WT cells (Figs. 1D and 3C).

However, we were unable to identify any candidate proteins that may contribute to the alternative pathway by transcriptome analysis of WT and DKO cells (data not shown). Long-term KD of NIPSNAP1 and 2 using shRNA did not result in the same phenotypes as those observed in KO cells. This discrepancy may be due to several reasons. First, induction of shRNA for longer than 2 weeks might be required to mimic KO cells. Indeed, KO cell lines are used in experiments at least 1–1.5 months after genome editing. Second, proteins are not completely downregulated by RNA interference compared with KO using the CRISPR/Cas9 system. In fact, 5% of NIPSNAP1 and 2 proteins were detectable in long-term shRNA-induced cells. These findings suggest that functional expression of a compensatory factor(s) and a salvage pathway for NIPSNAP1 and 2 are tightly regulated.

We demonstrated previously^7^ and in this study that NIPSNAP1 or 2 KD cells exhibited reduced cytokine production and altered mitochondrial bioenergetic profiles. These observations suggest that NIPSNAP1 and 2 act as a heterodimer, which is consistent with the results of a co-immunoprecipitation experiment^2^. However, it cannot be excluded that under stressed conditions, NIPSNAP1 and 2 also have the same function (i.e., both act as selective autophagy receptors of mitochondria)^3^. Thus, NIPSNAP1 and 2 might have different functions depending on the cellular conditions. A future study is required to identify alternative factors that complement NIPSNAP functions, elucidate the underlying mechanisms, and characterize these functions.

Inhibition of mitochondrial function by chemicals significantly reduced IL-8 induction by LPS, similar to the effect of transient depletion of NIPSNAP1 or 2 expression (Figs. 1B, 2G, and 4C). Although expression of NIPSNAP1 and 2 decreased under mitochondrial stress, expression of NIPSNAP3 did not (Fig. 2F). These results are consistent with the finding that the interaction of p62 with NIPSNAP3 was weaker than that with NIPSNAP1 and 2 (Fig. 2B). These functional differences are likely dependent on their domain structure; NIPSNAP1 and 2 have one NIPSNAP domain in their C-termini, while NIPSNAP3 has two such domains^1^. Chemically-induced mitochondrial stress leads to degradation of NIPSNAP1 and 2 via p62, which binds to a common sequence of NIPSNAP1 and 2 excluding the NIPSNAP domain. Moreover, p62 is involved in NF-κB activation via interactions with TIR domain-containing adapter-inducing interferon-β (TRIF) and receptor-interacting protein (RIP)^9^. IL-8 induction by LPS seemed to be augmented in p62 KD cells (Fig. 2C,D), suggesting that the interaction between p62 and NIPSNAP1 or 2 regulates mitochondrial quality control.

TLR4 signaling induces translocation of NF-κB into the nucleus, regulates transcription factors, and stimulates production of pro-inflammatory cytokines, such as IL-8 and IL-6^13^. Notably, among TLRs, only TLR4 stimulation induces NF-κB activation via both myeloid differentiation factor 88 (MyD88)- and TRIF-dependent pathways. We previously showed that NIPSNAP1 and 2 KD in T24 cells reduce production of IL-8 and IL-6 (MyD88-dependent cytokines) and C–C motif chemokine ligand 5 (CCL5, TRIF-dependent cytokine) upon TLR4 activation using LPS^7^. In addition, reactive oxygen species (ROS) are related to activation and repression of NF-κB signaling depending on the conditions. Mitochondrial dysfunction induces several stress responses, including ROS production^12^. In lung epithelial cells (the type II alveolar cell line C10), H_2_O_2_-mediated ROS might have an inhibitory effect on NF-κB activation^14^. However, different results have been reported in other cell types (the type II pulmonary epithelial cell line A549)^15^. Moreover, not only NF-κB but also AP-1 and other transcription factors are involved in TLR4-mediated IL-8 induction^16^. Although the relationship between IL-8 production and mitochondrial activity has not been investigated, we revealed that suppression of TLR4-meditated IL-8 production is attributable to mitochondrial hypoactivity upon temporary depletion of NIPSNAP1 or 2. Thus, NIPSNAP1 and 2 may be involved in induction of various cytokines through the MyD88- and TRIF-dependent pathways via mitochondrial quality control.

Macrolides are readily taken up and accumulate in alveolar cells. The concentration of CAM in alveolar cells is higher than 300 μg/mL after oral administration of 500 mg of CAM twice per day for 3 days^17^. By contrast, the concentration of CAM in serum is 4 μg/mL. Another study observed similar pharmacokinetics of CAM^18^; its concentration reached 700 μg/mL in alveolar cells. We found that CAM reduced the OCR in a dose-dependent manner, starting at a concentration of 100 μg/mL. Gupta et al. showed that treatment with CAM at a concentration of 15 μM (~ 11 μg/mL) for 16 h reduces the maximal respiration of mitochondria in the leukemic cell line K562^19^. These results imply that CAM temporarily suppresses mitochondrial bioenergetic homeostasis. Although mitochondrial stress inducers reduced the protein levels of NIPSNAP1 and 2, CAM did not (Fig. 5G). This suggests that mitochondrial stress induced by CAM is not as strong as that induced by general mitochondrial function inhibitors, such as CCCP (Fig. 2F). CAM might directly influence mitochondrial homeostasis, including the respiratory rate, by directly binding to NIPSNAP1 and 2 and inhibiting their functions. The reduction of mitochondrial activity underlies suppression of TLR4-mediated IL-8 production.

**Figure 6:**
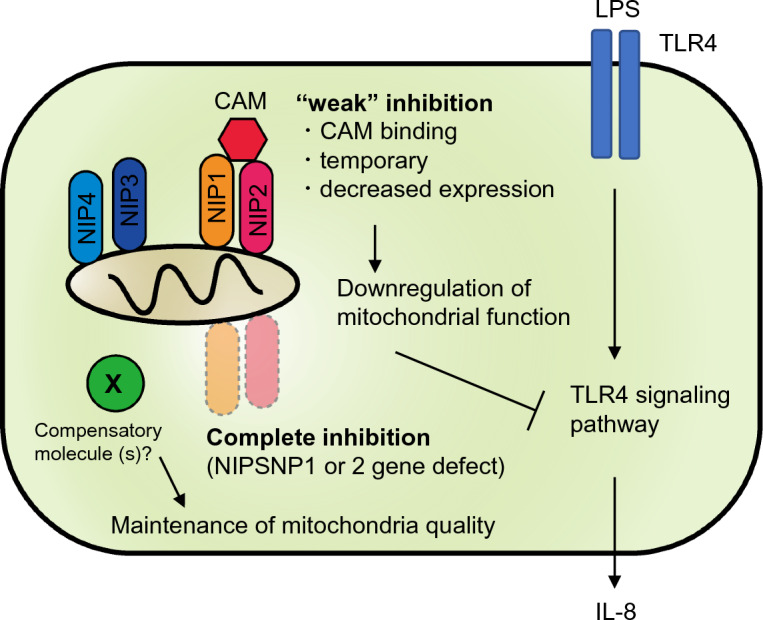
Graphical summary of the relationship between TLR4-mediated IL-8 production and NIPSNAP1- and 2-related mitochondrial homeostasis. IL-8 is induced by the binding of LPS to TLR4 followed by activation of TLR4 signaling pathways such as the NF-kB and AP-1 signaling pathways. CAM can interact with mitochondrial NIPSNAP1 and 2 (NIP1 and 2). Temporary and weak inhibition of NIPSNAP1 and 2 by CAM binding perturbs mitochondrial quality control, followed by suppression of IL-8 induction. Mitochondrial function and IL-8 production are maintained upon complete depletion (i.e., KO) of NIPSNAP1 and 2, suggesting that "molecular X", an alternative protein to NIPSNAP1 and 2, compensates and maintains mitochondrial homeostasis.

Overall, we concluded that treatment with CAM leads to temporary functional inhibition of NIPSNAP1 and 2 by binding to these proteins, followed by a decline in mitochondrial function. Mitochondrial hypoactivity underlies the reduction of TLR4-mediated IL-8 production in airway epithelial cells (Fig. 6). However, we could not identify the alternative factor (shown in Fig. 6 as X) of NIPSNAP1 and 2 or its mechanism by performing bulk RNA sequencing analysis of KO cells (data not shown). To overcome this hurdle, multi-omics analyses, such as proteomics and metabolomics analyses, and mitochondrial dynamics analyses under stressed conditions should be performed in future studies. Finally, cytokine production via the TLR-mitochondria axis seems to be tightly regulated. Elucidation of the underlying molecular mechanism will facilitate the development of next-generation immunomodulatory drugs and increase understanding of the immune system.

## Methods

Information about LPS and CAM was described previously^7^. DFP (379409) was purchased from Sigma-Aldrich (St. Louis, MO, USA) and dissolved in sterile water. CCCP (304-16993) and Q-VD-OPH (S7311) were obtained from FUJIFILM Wako Pure Chemical Corp. (FUJIFILM Wako, Osaka, Japan) and Selleck Chemicals (Houston, TX, USA), respectively, and dissolved in dimethyl sulfoxide.

### Antibodies

Mouse anti-FLAG (014-22383) and anti-β actin (281-98721, FUJIFILM Wako), anti-p62 (PM045), and rabbit anti-FLAG (PM020; Medical and Biological Lab., Tokyo, Japan), anti-NIPSNAP1 (D1Y6S; Cell Signaling Technology, Danvers, MA, USA), anti-NIPSNAP2 (LS-C337922; LifeSpan BioSciences, Seattle, WA, USA), and anti-NIPSNAP3 (11,789-I-AP; Proteintech, Rosemont, IL, USA) antibodies were purchased from the respective manufacturers. A rabbit anti-HSP60 antibody was kindly gifted by Dr. Hideaki Itoh^20^. All western blotting (WB) images were developed using the iBright FL1000 imaging system (Thermo Fisher Scientific, Waltham, MA, USA). These blots were normally developed without overexposure. Overexposed images are labeled “overexposure” at the bottom.

### Cell culture

The culturing method of BEAS-2B cells was described previously^7^. H1-HeLa cells (CRL-1985) were obtained from American Type Culture Collection (Manassas, VA, USA) and maintained in DMEM-high glucose supplemented with 10% (v/v) fetal bovine serum, 100 U/mL penicillin G, and 10 μg/mL streptomycin (FUJIFILM Wako). siRNAs against NIPSNAP1 (no. 1, s16166 (sense, 5′-GAAUGGGUCCCAACAUCUAtt-3′ and anti-sense, 5′-UAGAUGUUGGGACCCAUUCtg-3′); no. 2, s16167 (sense, 5′-GAUCCAGUUUCACAAUGUAtt-3′ and anti-sense, 5′-UACAUUGUGAAACUGGAUCtt-3′); and no. 3, s16168 (sense, 5'-CCAUCUCUGGGCCUAUAAAtt-3′ and anti-sense, 5′-UUUAUAGGCCCAGAGAUGGtg-3′)), NIPSNAP2 (no. 1, s5614 (sense, 5′-CAGUUCCACUUAUUCAGGAtt-3′ and anti-sense, 5′-UCCUGAAUAAGUGGAACUGtg-3′); no. 2, s5615 (sense, 5′-GGAAUUUCGUAAGGCAAGAtt-3′ and anti-sense, 5′-UCUUGCCUUACGAAAUUCCaa-3′); and no. 3, s5616 (sense, 5′-CCUAGAAGCAUACAACAAAtt-3′ and anti-sense, 5′-UUUGUUGUAUGCUUCUAGGca-3′)), and p62 (s16961) were purchased from Thermo Fisher Scientific. Cells were transfected with these siRNAs using RNAiMAX (Thermo Fisher Scientific) according to the manufacturer's instructions. After incubation for 48 h, the level of human IL-8 in the supernatant was measured using the Duoset ELISA development system (R&D Systems, Minneapolis, MN, USA). After LPS stimulation, cells were trypsinized and counted using trypan blue for normalization.

### Generation of NIPSNAP1 and 2 KO cells

sgRNAs against NIPSNAPs were designed using the optimized CRISPR Design tool of Dr. Zhang's laboratory at Massachusetts Institute of Technology (Cambridge, MA, USA) (this tool is no longer available). sgRNAs against NIPSNAP1 (guide A: 5′-GAGCCATGTTGGAGCCGCAA-3′ and guide B: 5'-GCATCTCTGTGACGGCGCGG-3') and NIPSNAP2 (guide A: 5′-GTCTTCTCGAGATCTGTTGC-3′ and guide B: 5′-GGCTAAAATCCTTATTTGTC-3′) were synthesized by Fasmac Co., Ltd. (Atsugi, Japan). Annealed guide A and B oligonucleotides were inserted into the pSpCap9n(BB)-2A-GFP (PX461) and pSpCap9n(BB)-2A-puro (PX462) plasmids (Addgene, Watertown, MA, USA), respectively. These plasmids were transfected using Lipofectamine 3000 (Thermo Fisher Scientific) into monoclonal BEAS-2B cells (parental) because BEAS-2B cells comprise various cell types such as basal and goblet cells. After 24 h, transfected cells were selected by treatment with 1 μg/mL puromycin for 2 days. Monoclones were established from 96-well plates after limiting dilution. Knockout of proteins was confirmed by WB or genome sequencing.

### Generation of tetracycline-inducible shRNA expression cells

The sequences of shRNAs described below were obtained from the database of MISSON® shRNA (Sigma-Aldrich). shRNAs against NIPSNAP1 (5′-GATCCAGTTTCACAATGTAAA-3′) and NIPSNAP2 (5′-GTGTTGCCAAAGATTCACGAA-3′) were cloned into Tet-PLKO-puro (Addgene; 21915) according to Addgene’s protocol. The Tet-PLKO-puro plasmid was used as a control. Supernatants containing shRNA-encoding lentiviruses were collected, and the copy number was quantified using a qPCR Lentivirus Titer Kit (Applied Biological Materials Inc., British Columbia, Canada). Then, lentiviruses were transduced into BEAS-2B cells at a multiplicity of infection of 5. shRNA-integrated cells were selected by culture in maintenance medium (LHC9 medium containing 0.5 μg/mL puromycin, 100 U/mL penicillin, and 100 μg/mL streptomycin) for 1 week. Then, cells were maintained using maintenance medium or cultured in medium supplemented with 100 ng/mL DOX (FUJIFILM Wako) for 2 weeks, during which time the medium was changed every 2 days.

### Immunoprecipitation

cDNAs of C-terminally FLAG-tagged NIPSNAP1, 2, and 3B were cloned into the pCAGGS plasmid. pEGFP-C1 was obtained from Takara Bio (Shiga, Japan). BEAS-2B cells were transfected with these plasmids using Lipofectamine 3000 and incubated for 48 h with 10 μM Q-VD-OPH to prevent induction of apoptosis by transfection. After incubation for 24 h, cells were lysed with cell lysis buffer [1% (v/v) Triton-X 100, 0.5% (w/v) sodium deoxycholate, 0.15 M NaCl, 2 mM EDTA, and 50 mM HEPES–NaOH 7.4] supplemented with a protease inhibitor cocktail (25955-11; Nacalai Tesque, Kyoto, Japan). The lysates were reacted with anti-FLAG tag antibody-conjugated beads (018-22783, FUJIFILM Wako) overnight at 4 °C with rotation. After four washes with cell lysis buffer, immunoprecipitated proteins were eluted in 1 × SDS sample buffer by boiling.

### Immunofluorescence

BEAS-2B cells were transfected with the FLAG-tagged NIPSNAP1, 2, or 3 expression plasmid using Lipofectamine 3000 (Thermo Fisher Scientific). At 24 h after transfection, cells were passaged on a collagen-coated glass bottom dish (Matsunami Glass Ind., Ltd., Osaka, Japan) and incubated for 24 h. Cells were fixed by incubation in 4% (v/v) paraformaldehyde at room temperature for 15 min. Furthermore, specimens were treated with 1 × PBS (-) containing 5% (v/v) normal donkey serum (Abcam, Cambridge, UK) and 0.3% (v/v) Triton-X 100 for 1 h following washing with 1 × PBS (-). A rabbit anti-p62 or anti-HSP60 antibody and a mouse anti-FLAG antibody were diluted using 1 × PBS (-) containing 1% (w/v) bovine serum albumin and 0.3% Triton-X 100, and samples were incubated with the diluted antibodies overnight at 4 °C. After rising with 1 × PBS (-), specimens were reacted with Alexa Fluor 488- or 647-conjugated donkey anti-rabbit IgG (Thermo Fisher Scientific) and 4′,6-diamino-2-phenylindole at room temperature for 1 h. Finally, specimens were mounted with 2% 1,4-diazabicyclo[2.2.2]octane (Sigma-Aldrich) after six washes with 1 × PBS (-). Fluorescent images were obtained using a LSM780 microscope (Carl Zeiss Microscopy, Jena, Germany) and cropped using Adobe Photoshop software (Adobe, San Jose, CA, USA).

### Quantitative PCR

To calculate the KD efficiency, total RNA was purified using a RNeasy Plus Kit (Qiagen, Venlo, Netherlands). Then, cDNA was synthesized using ReverTra Ace qPCR RT Master Mix with gDNA remover according to the manufacturer’s protocol (TOYOBO, Osaka, Japan). SYBR Green-based quantitative PCR was performed using KOD SYBR qPCR Mix (TOYOBO) and LightCycler 480 System II (Roche, Basel, Switzerland). The following primers were used: NIPSNAP1-forward, 5'-CTGCTCCTCGAGTTCAGCTT-3'; NIPSNAP1-reverse, 5'-CAGAGGCGAGATCTTCAAGG-3'; NIPSNAP2-forward, 5'-CCAGGAAGAATCAGCTCCTG-3'; NIPSNAP2-reverse, 5'-TCCCTGTAAGCCCAAAGATG-3'; human β-actin-forward, 5'-AGATGGCCACGGCTGCT-3; and human β-actin-reverse, 5'-AACCGCTCATTGCCAATGG-3'. Relative mRNA expression was calculated using the 2^–∆∆Ct^ method.

### Measurement of the mtDNA copy number

After drug treatment, cells were washed with 1 × PBS (-) and then the genome was purified using DNeasy Blood and Tissue Kits (Qiagen). The copy number of mtDNA was measured using a Human Mitochondrial DNA Monitoring Primer set (Takara Bio) according to the manufacturer's instructions.

### Flex analyzer

The day before siRNA transfection, BEAS-2B cells were seeded into a 12-well plate at a density of 1.0 × 10^5^ cells per well. After incubation overnight, cells were transfected with siRNAs against NIPSNAP1 and 2 using RNAiMAX. The next day, 2.0 × 10^4^ cells were re-seeded into a homemade collagen-coated Agilent Seahorse XF96 Cell Culture Microplate (101085-004; Agilent Technologies, Santa Clara, CA, USA) and then incubated overnight. To measure the OCR and ECAR after CAM treatment, 2.0 × 10^4^ BEAS-2B cells were seeded into a homemade collagen-coated Agilent Seahorse XF96 Cell Culture Microplate. After incubation for 8 h, CAM was added to each well and cells were further incubated overnight. The mitochondrial OCR and ECAR were measured using Extracellular Flux Assay Kits and a Seahorse XF Cell Mito Stress Test kit (103015-100, Agilent Technologies) with the Seahorse XFe96 analyzer (Agilent Technologies) according to the manufacturer's instructions. During the measurement, oligomycin, carbonyl cyanide-*p*-trifluoromethoxyphenylhydrazone (FCCP), and a mixture of rotenone and antimycin A were sequentially injected at final concentrations of 1, 1, and 2 μM, respectively. The bioenergetic profiles were calculated using Wave software (Ver. 2.6.3; Agilent Technologies).

### Imaging and statistical analyses

All graphs were generated using Prism v.9 (GraphPad Software, San Diego, CA, USA). Statistical analyses were conducted using the Student's t-test or a one-way ANOVA. A difference with a p-value of less than 0.05 was considered significant. Analyzed data are presented as the mean ± standard deviation (SD).

### Supplementary Information

Supplementary Information.

## Data Availability

The data that support the findings of this study are available from the corresponding author, upon reasonable request.
