# Supplementary material for: The clarithromycin-binding proteins NIPSNAP1 and 2 regulate cytokine production through mitochondrial quality control
Source: Sci Rep. 2024 Jan 29;14:2354. doi: 10.1038/s41598-024-52582-7 (PMC10824736; doi:10.1038/s41598-024-52582-7)
Supplement: Supplementary file 1 — Supplementary Information. [file 41598_2024_52582_MOESM1_ESM.pdf]

Supplemental Information

The clarithromycin-binding proteins NIPSNAP1 and 2 regulate cytokine production through mitochondrial quality control.

Soh Yamamoto, Noriko Ogasawara, Yukari Mitsuhashi, Kenichi Takano, and Shin-ichi Yokota

Supplemental Figure S1.
Uncropped western blotting images (the left or middle panels) and PVDF membrane images (the right images) of Figures 1A, 1C, 2B, 2C, 2F, 4B, and 5G.

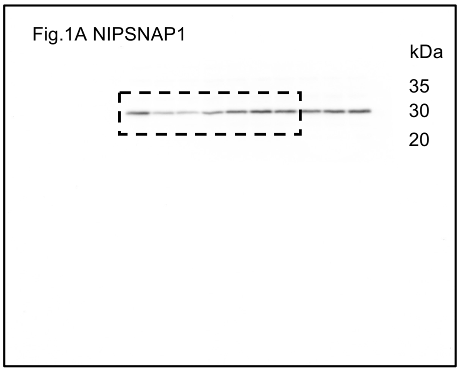

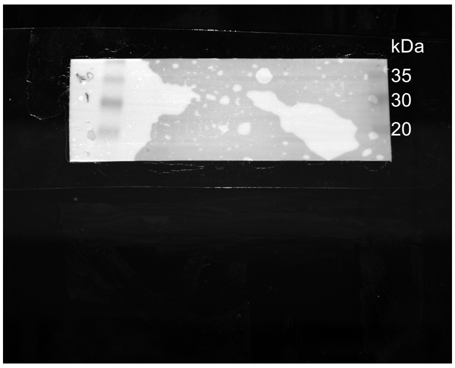

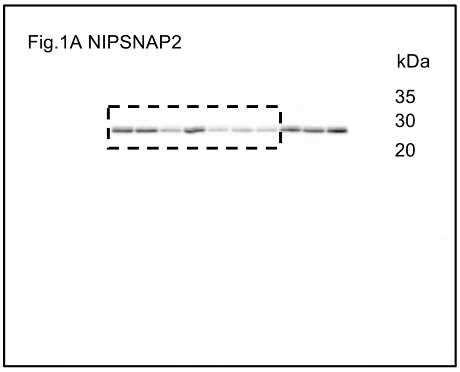

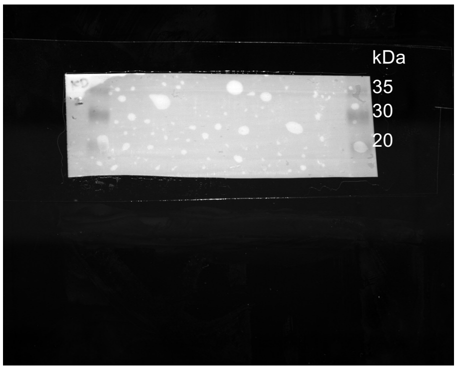

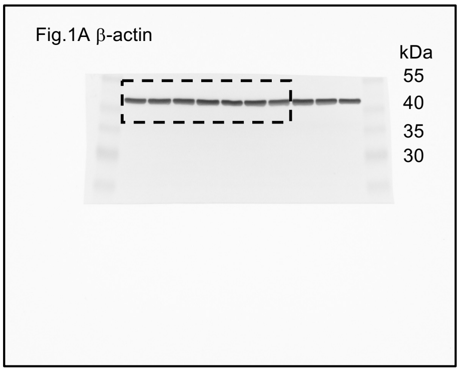

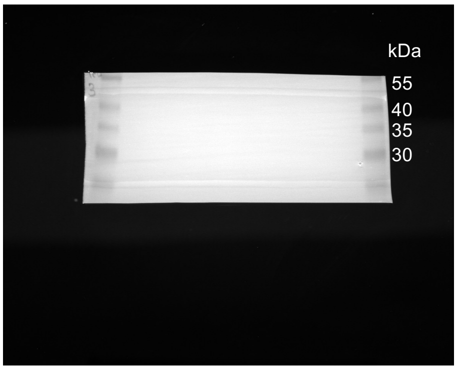

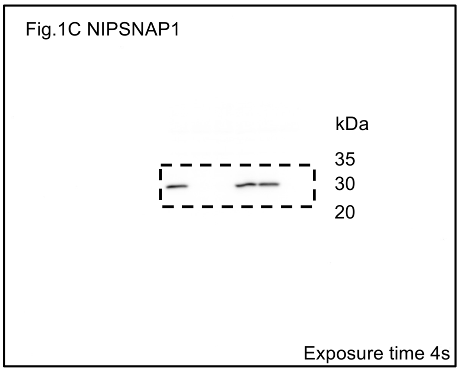

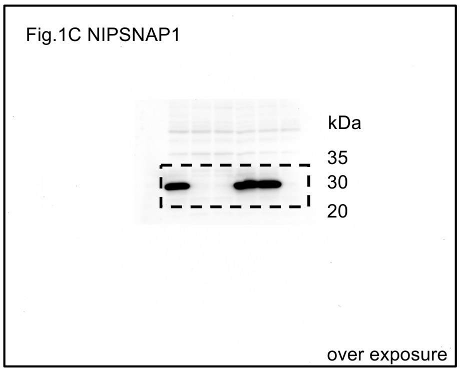

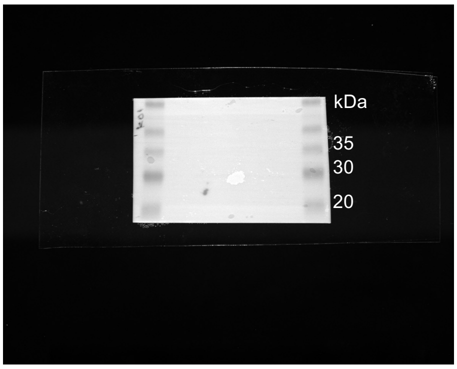

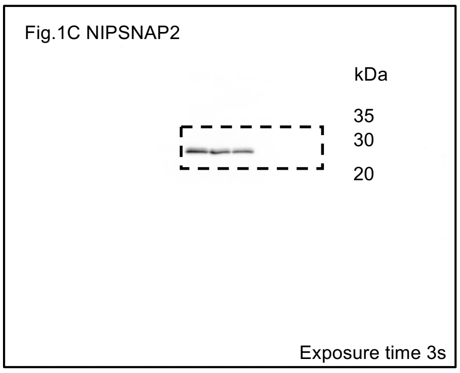

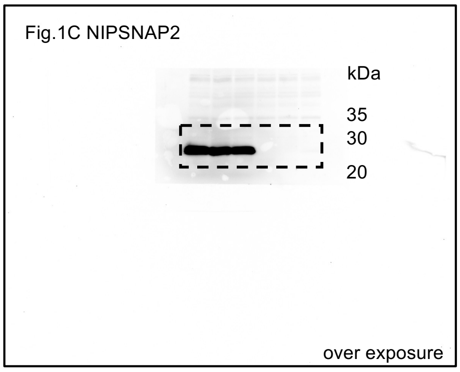

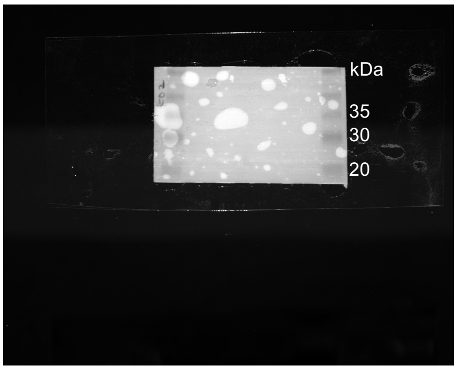

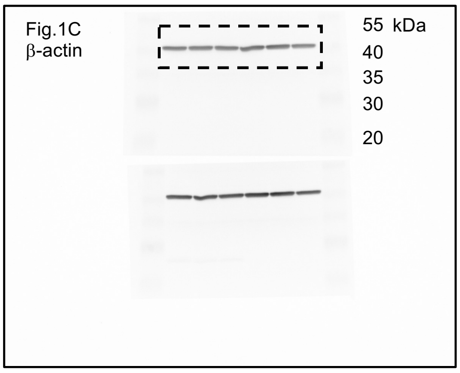
Fig.1C
β-actin
55 kDa
40
35
30
20

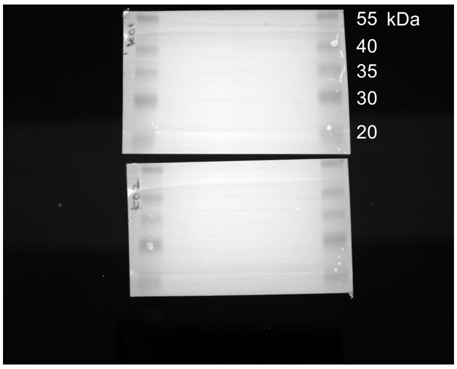
55 kDa
40
35
30
20

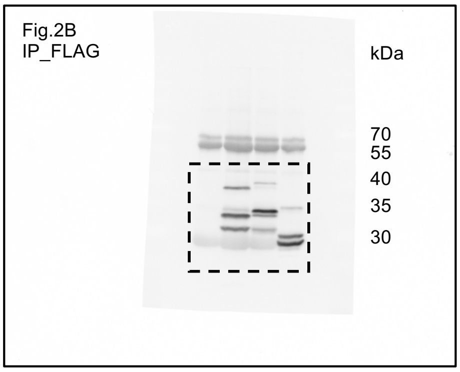
Fig.2B
IP_FLAG
kDa
70
55
40
35
30

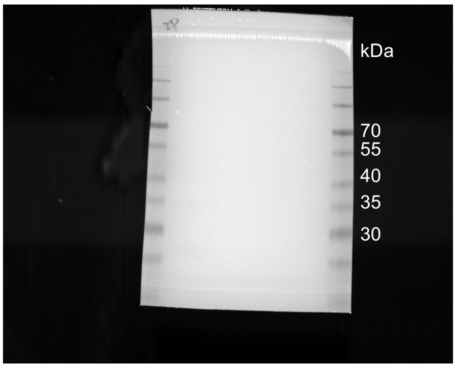
kDa
70
55
40
35
30

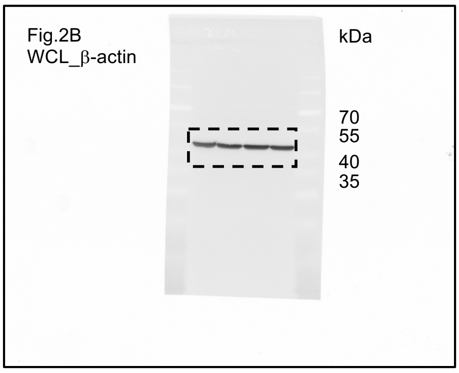
Fig.2B
WCL_β-actin
kDa
70
55
40
35

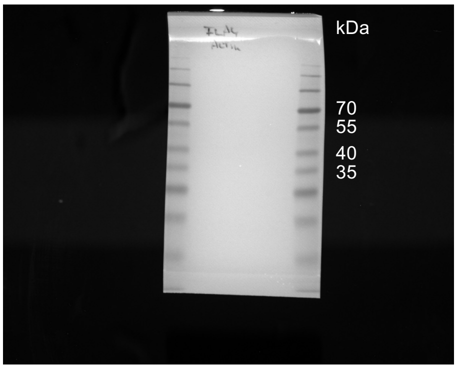
kDa
70
55
40
35

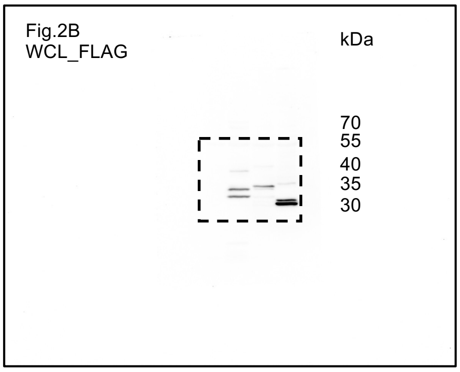
Fig.2B
WCL_FLAG
kDa
70
55
40
35
30

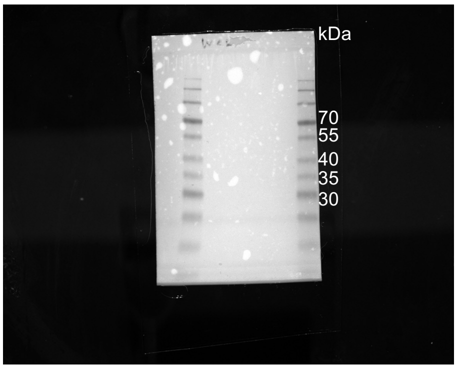
kDa
70
55
40
35
30

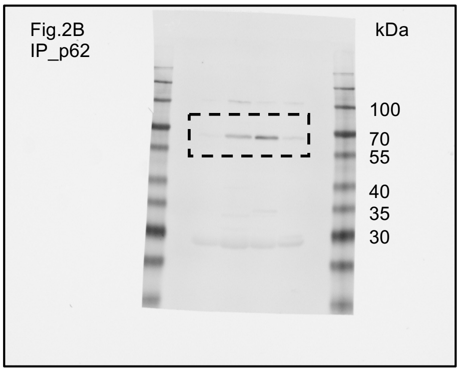
Fig.2B
IP_p62
kDa
100
70
55
40
35
30

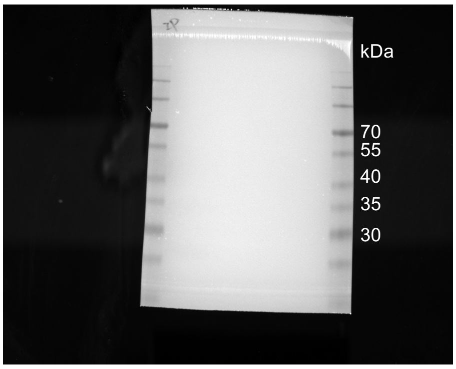
kDa
70
55
40
35
30

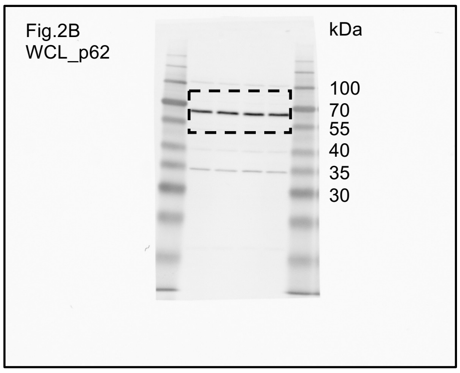
Fig.2B
WCL_p62
kDa
100
70
55
40
35
30

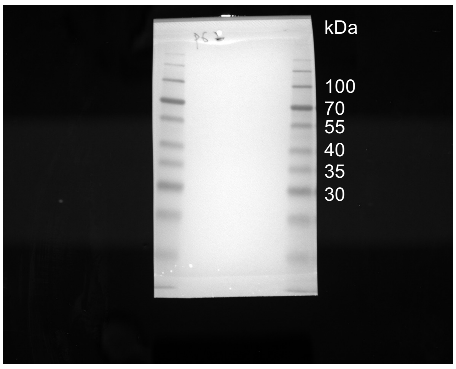
kDa
100
70
55
40
35
30

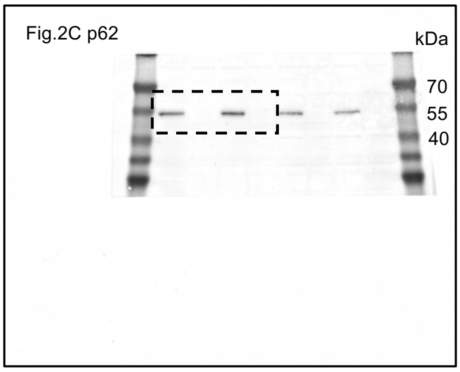
Fig.2C p62
kDa
70
55
40

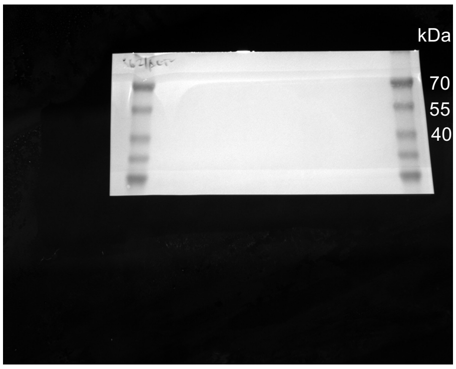
kDa
70
55
40

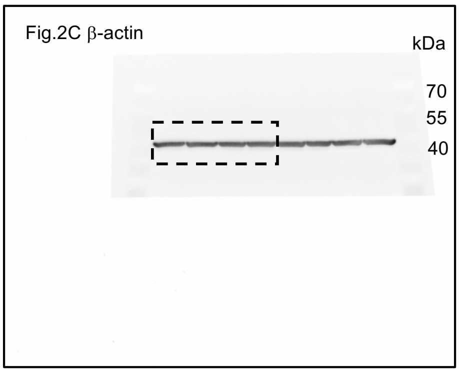
Fig.2C β-actin
kDa
70
55
40

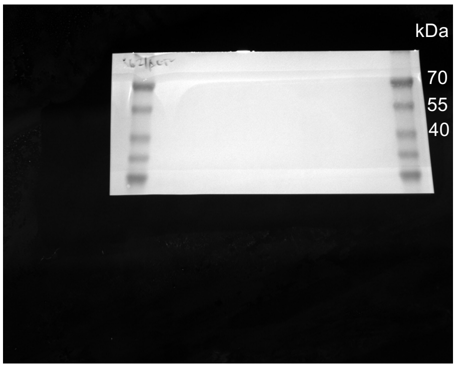
kDa
70
55
40

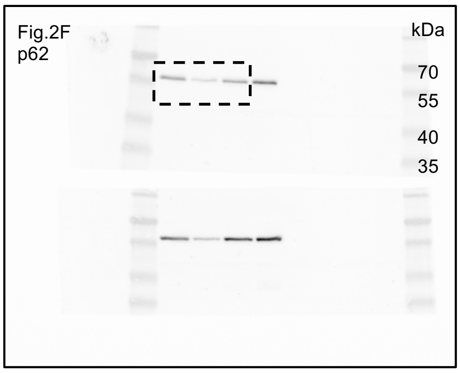
Fig.2F
p62
kDa
70
55
40
35

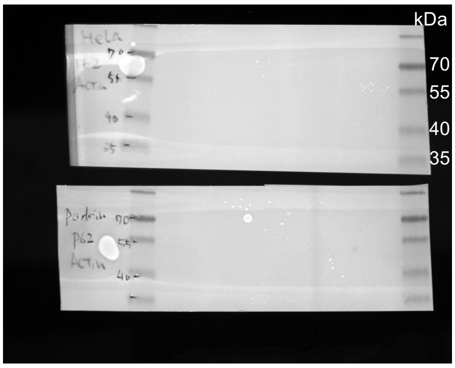
kDa
70
55
40
35

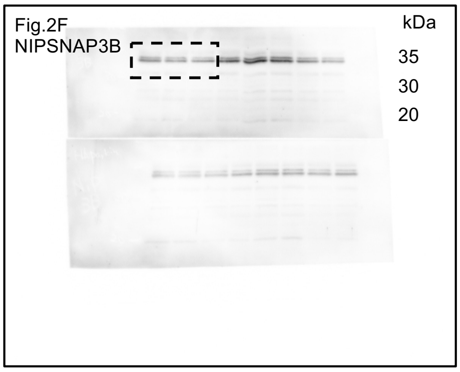
Fig.2F
NIPSNAP3B
kDa
35
30
20

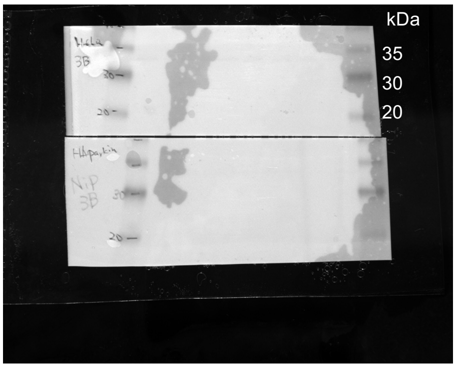
kDa
35
30
20

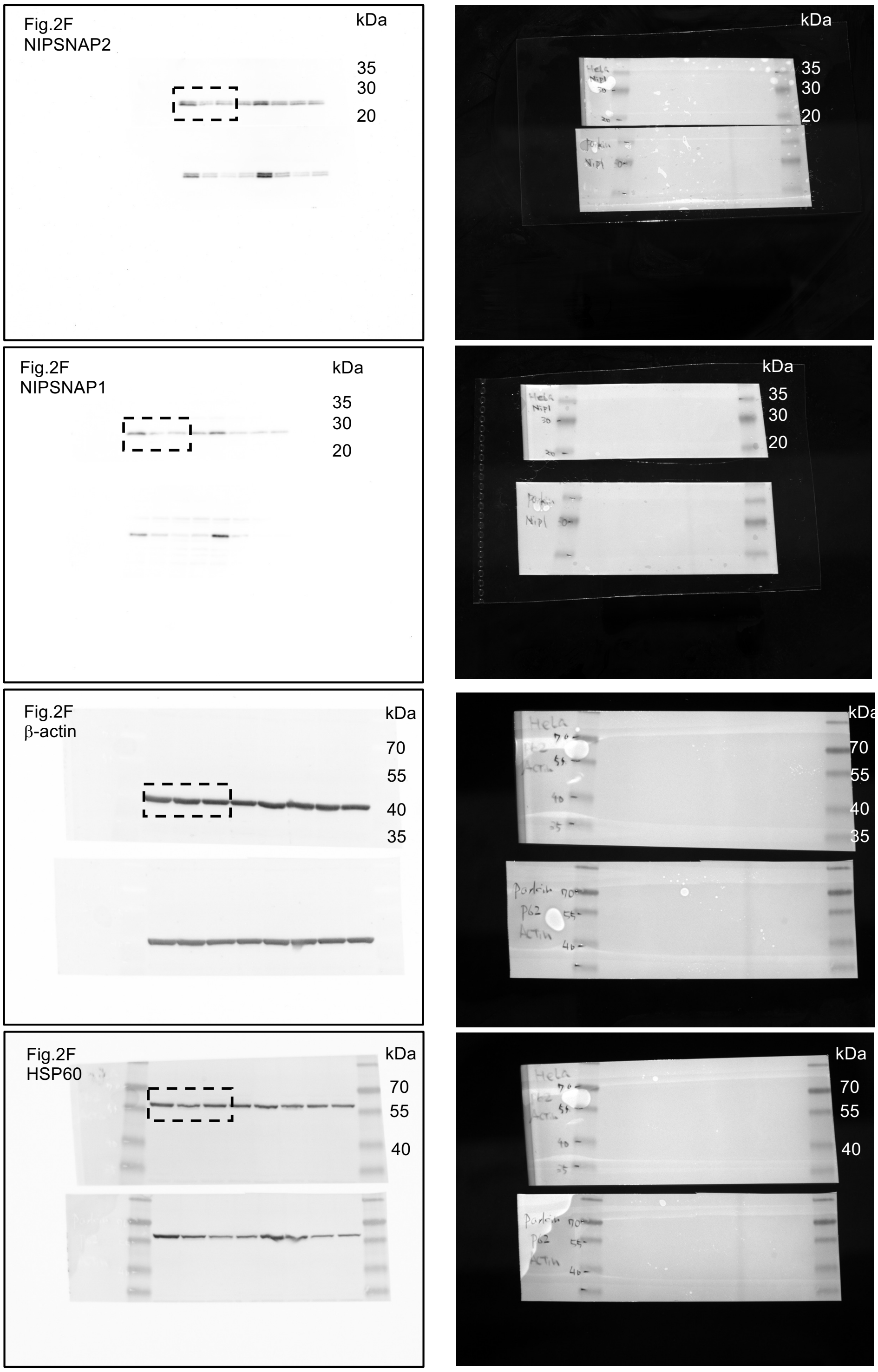
Fig.2F
NIPSNAP2
kDa
35
30
20
Fig.2F
NIPSNAP1
kDa
35
30
20
Fig.2F
β-actin
kDa
70
55
40
35
Fig.2F
HSP60
kDa
70
55
40

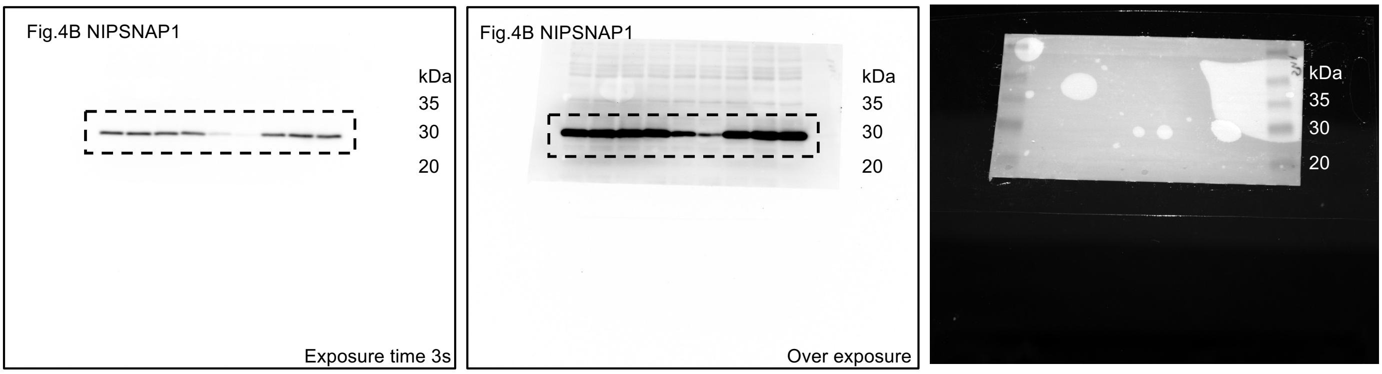
Fig.4B NIPSNAP1
kDa
35
30
20
Exposure time 3s
Fig.4B NIPSNAP1
kDa
35
30
20
Over exposure
kDa
35
30
20

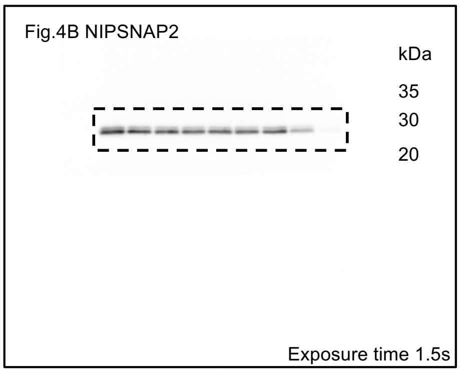
Fig.4B NIPSNAP2
kDa
35
30
20
Exposure time 1.5s

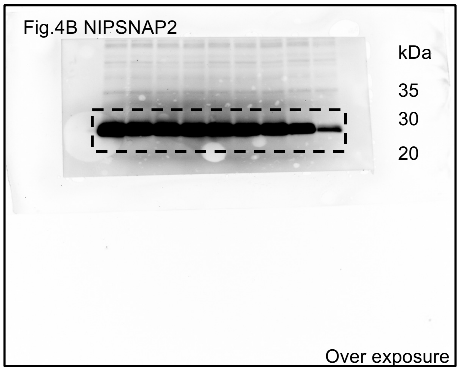
Fig.4B NIPSNAP2
kDa
35
30
20
Over exposure

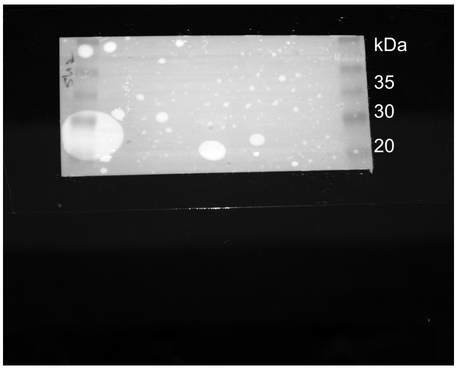
kDa
35
30
20

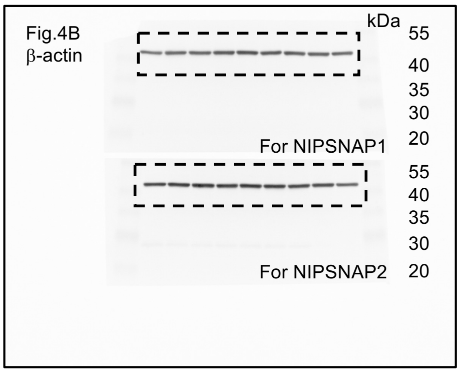
Fig.4B
β-actin
kDa
55
40
35
30
20
For NIPSNAP1
55
40
35
30
For NIPSNAP2
20

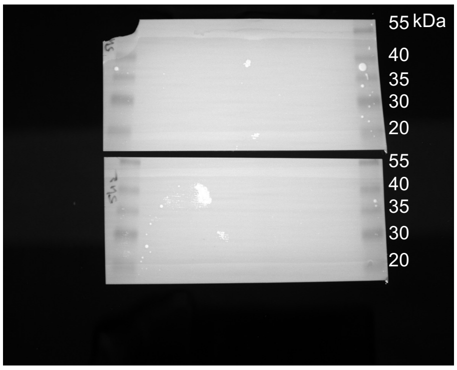
55 kDa
40
35
30
20
55
40
35
30
20

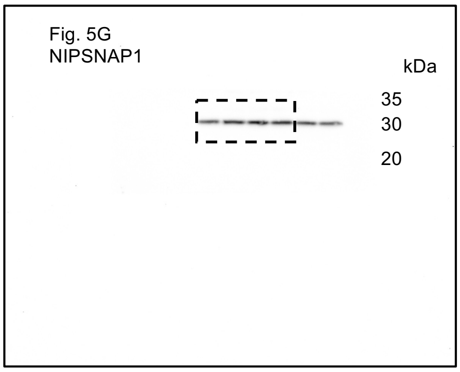
Fig. 5G
NIPSNAP1
kDa
35
30
20

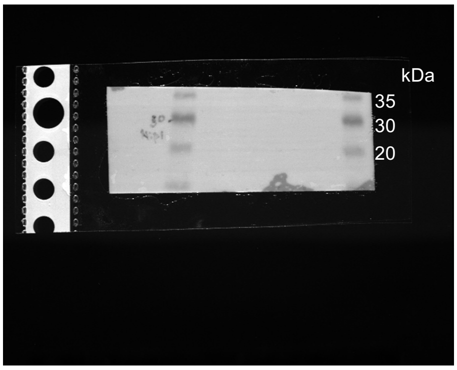
kDa
35
30
20

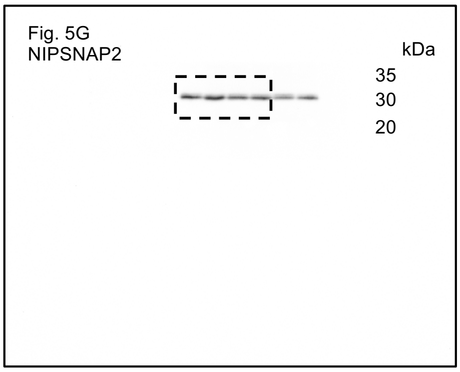
Fig. 5G
NIPSNAP2
kDa
35
30
20

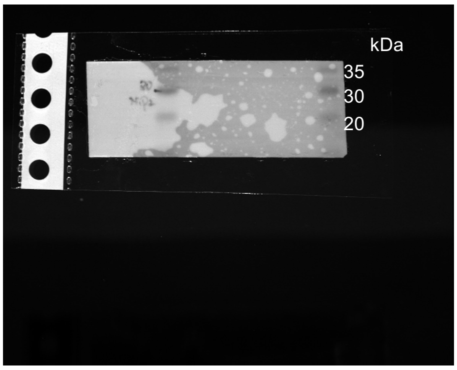
kDa
35
30
20

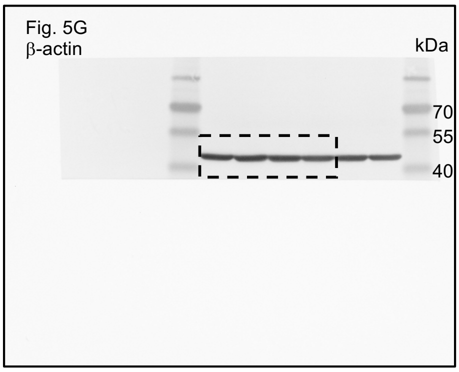
Fig. 5G
β-actin
kDa
70
55
40

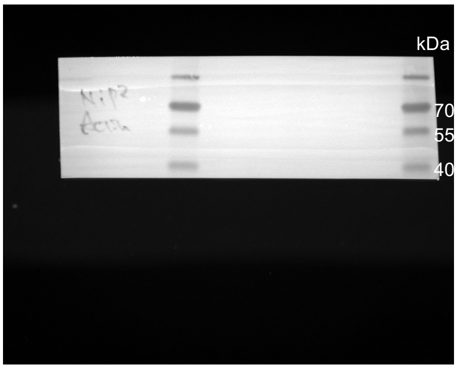
kDa
70
55
40
